# Novel deuterium metabolic imaging technique reveals distinct patterns of postprandial hepatic glucose homeostasis in individuals with type 1 diabetes and healthy control individuals: a case–control study

**DOI:** 10.1007/s00125-026-06677-7

**Published:** 2026-02-13

**Authors:** Alessandro Brunasso, Naomi F. Lange, Simone Poli, Michele Schiavon, David Herzig, Chiara Dalla Man, Roland Kreis, Lia Bally

**Affiliations:** 1 Department of Information Engineering, University of Padova, Padova, Italy; 2 Department of Visceral Surgery and Medicine, Inselspital, Bern University Hospital, University of Bern, Bern, Switzerland; 3 Graduate School for Health Sciences, University of Bern, Bern, Switzerland; 4 Magnetic Resonance Methodology, Institute of Diagnostic and Interventional Neuroradiology, University of Bern, Bern, Switzerland; 5 Translational Imaging Center, Sitem-Insel, Bern, Switzerland; 6 Graduate School for Cellular and Biomedical Sciences, University of Bern, Bern, Switzerland; 7 Department of Diabetes, Endocrinology, Nutritional Medicine and Metabolism (UDEM), Inselspital, Bern University Hospital, University of Bern, Bern, Switzerland

**Keywords:** Deuterium metabolic imaging, Endogenous glucose production, Glucose fluxes, Glucose metabolism, Glycogen, Insulin sensitivity, Liver glucose metabolism, Magnetic resonance spectroscopy, Stable isotopes, Type 1 diabetes

## Abstract

**Aims/hypothesis:**

Subcutaneous insulin delivery in individuals with insulin-deficient type 1 diabetes bypasses the portal circulation, disrupting the physiological porto-systemic insulin gradient and affecting postprandial hepatic glucose regulation. However, direct, non-invasive measurement of these liver-specific dynamics and their deviation from normal physiology in individuals with type 1 diabetes is challenging. To address this, we integrated metabolic imaging with whole-body tracer dilution to map postprandial glucose metabolism in both the liver and systemically in adults with type 1 diabetes and healthy control individuals.

**Methods:**

In this cross-sectional study, ten adults with type 1 diabetes and ten healthy control individuals with similar age, BMI and gender distributions were enrolled. After an overnight fast, participants ingested 60 g [6,6′-^2^H_2_]-glucose (D-Glc); subcutaneous insulin was administered to type 1 diabetes participants according to their carbohydrate-to-insulin ratio. Interleaved deuterium metabolic imaging (DMI) and ^13^C-magnetic resonance spectroscopy (^13^C-MRS) at 7 T were performed from pre-ingestion to 150 min post-ingestion to quantify hepatic D-Glc and glycogen concentrations. Blood samples were collected to measure plasma glucose, insulin and glucagon. Postprandial glucose–insulin dynamics were quantified using the single tracer oral minimal model, accounting for non-steady-state insulin exposure.

**Results:**

At baseline, individuals with type 1 diabetes had significantly higher plasma glucose concentrations than control individuals (10.7±2.3 and 5.2±0.4 mmol/l, respectively; *p*<0.001), while preprandial glycogen levels did not differ significantly. Following D-Glc administration, hepatic D-Glc increased more markedly in the individuals with type 1 diabetes compared with the control group (peak values 4.7±2.0 and 3.0±0.8 mmol/l, respectively; *p*=0.02). In the postprandial period, glycogen levels did not significantly rise at 150 min in type 1 diabetes, whereas a clear increase was observed in control individuals (iAUC_0–180_=2.4 mol/l × min). Despite similar systemic insulin exposure and no significant differences in postprandial glucagon concentrations between groups, individuals with type 1 diabetes demonstrated significantly reduced suppression of endogenous glucose production (*p*=0.001) but similar insulin-dependent glucose disposal. Hierarchical clustering identified two distinct type 1 diabetes subgroups: Subgroup 1 exhibited a steeper increase in both hepatic and systemic D-Glc profiles, while subgroup 2 showed a divergent D-Glc trajectory and net glycogen depletion relative to accumulation in subgroup 1 (iAUC_0–180_=−3.0 vs 2.5 mol/l × min, *p*=0.04), despite no overt clinical differences between subgroups.

**Conclusions/interpretation:**

By integrating DMI/^13^C-MRS liver imaging with systemic stable-isotope modelling, this comparative study demonstrates significantly altered hepatic glucose metabolism in adults with well-managed type 1 diabetes vs control individuals, together with substantial phenotypic heterogeneity within the type 1 diabetes cohort. These findings highlight the potential of non-invasive metabolic phenotyping to resolve metabolic alterations and inter-individual variation in type 1 diabetes, which are essential steps towards the provision of precision medicine.

**Graphical Abstract:**

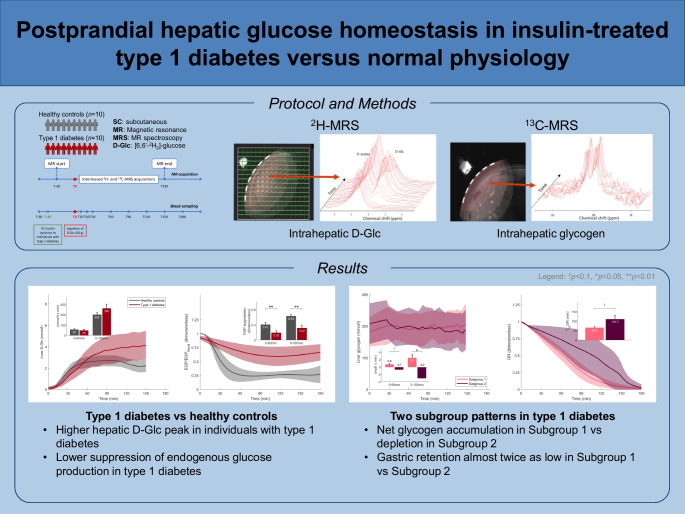

**Supplementary Information:**

The online version of this article (10.1007/s00125-026-06677-7) contains peer-reviewed but unedited supplementary material.


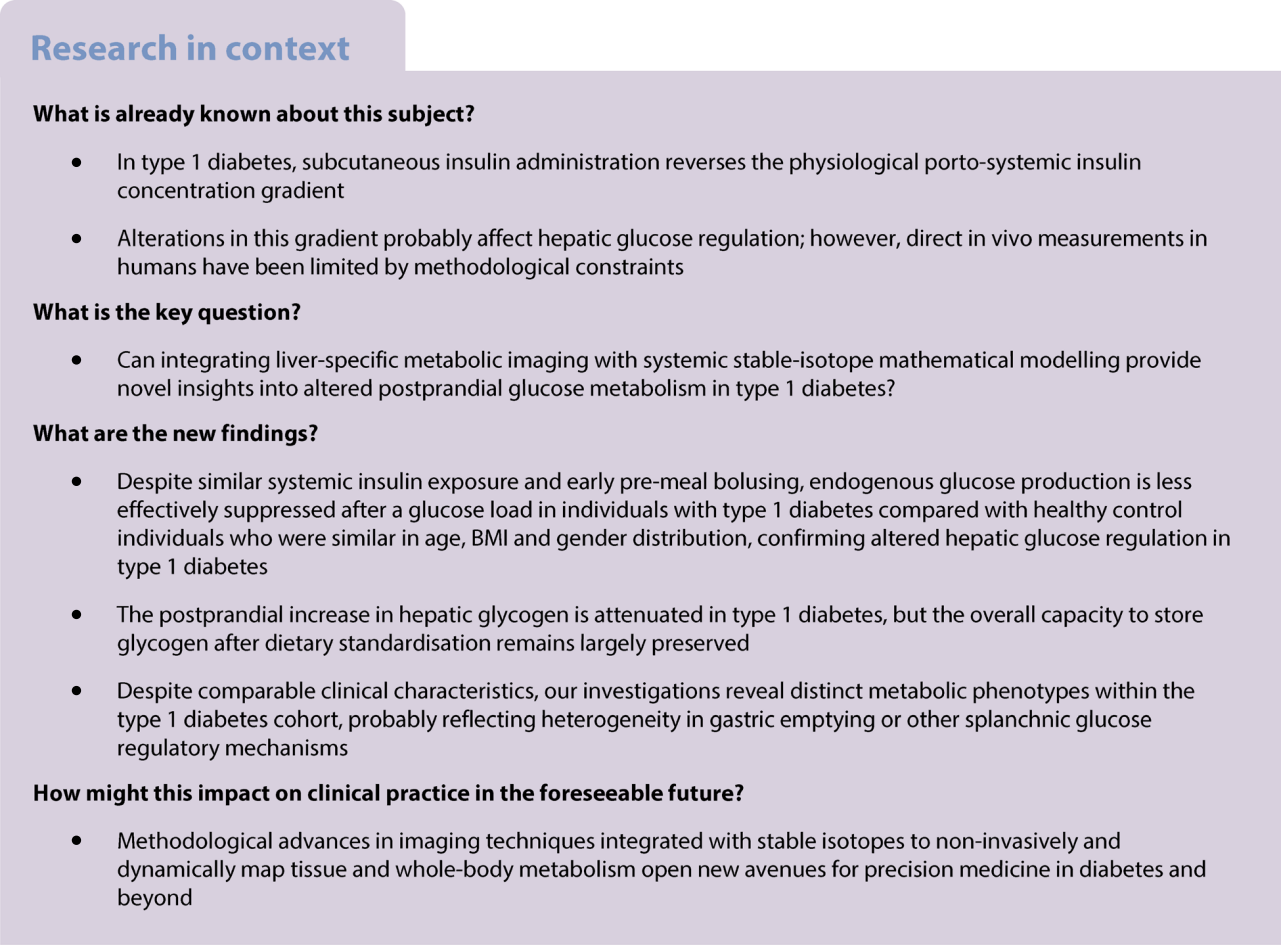


## Introduction

Type 1 diabetes is characterised by the absolute loss of endogenous insulin production, and hence dependence on lifelong subcutaneous (SC) insulin administration [1, 2]. Unlike endogenous insulin, which enters the portal circulation and creates a porto-systemic insulin gradient, SC delivery bypasses the portal vein and exposes the liver to relatively less insulin than peripheral tissues [3–6]. The absence of an insulin gradient alters hepatic glucose regulation following meals, predisposing individuals with type 1 diabetes to inadequate suppression of endogenous glucose production and impairment of hepatic glucose uptake [4, 5]. Collectively, these factors can compromise postprandial glucose management, which is a persistent challenge in type 1 diabetes management, even among individuals who are achieving recommended HbA_1c_ targets [7].

Investigating metabolic aspects of hepatic glucose regulation in vivo in humans remains methodologically challenging. While ^18^F-fluorodeoxyglucose positron emission tomography [8] offers a non-invasive approach to quantify glucose uptake, the route of tracer does not represent the physiological route of ingestion, and the method involves radiation exposure and does not provide insights into the conversion of intrahepatic glucose into glycogen, thereby challenging its applicability for the investigation of postprandial metabolism in humans [3, 4, 9]. Deuterium metabolic imaging (DMI) [10–18] has emerged as a promising alternative for organ-specific metabolic profiling, including in the liver. By tracking orally or intravenously administered ^2^H-labelled glucose, DMI enables real-time, liver-specific quantification of glucose kinetics, but does not allow detection of ^2^H-labelled glycogen [19]. We have recently developed a protocol that integrates DMI with ^13^C magnetic resonance spectroscopy (MRS) [20, 21], enabling visibility of the C_1_ carbon of glycogen and its quantification at natural abundance [13].

In this study, we combine this advanced imaging protocol with whole-body tracer dilution techniques [22] to compare postprandial glucose metabolism at both the hepatic and systemic levels in adults with type 1 diabetes and healthy control individuals. This comprehensive approach provides quantitative insights into metabolic alterations associated with type 1 diabetes pathophysiology, including those arising from non-physiological SC insulin administration.

## Methods

### Study design and population

This cross-sectional study was conducted at the University Hospital Bern and the Translational Imaging Center at Sitem-Insel. Ethical approval was obtained from the Ethics Committee Bern (2019-02346), and all participants provided written informed consent before any study-related activity. We enrolled ten adults with type 1 diabetes from the local outpatient clinic, with a diabetes duration ≥2 years or biochemical evidence of absolute insulin deficiency (C-peptide <100 pmol/l with concurrent plasma glucose ≥4 mmol/l). Individuals with type 1 diabetes were required to have an HbA_1c_ ≤64 mmol/mol (8%) and to be on functional insulin therapy, using either multiple daily injections or insulin pump therapy. The control group comprised ten healthy adults with no chronic diseases or regular medication use, who were selected to be similar in age, BMI and gender distribution to the type 1 diabetes group using a prospective distribution-based enrolment strategy. Race or ethnicity data were not specifically collected.

Exclusion criteria included any diabetes aetiology other than type 1 (for the diabetes group), pregnancy or breastfeeding, use of medications affecting glucose metabolism (except SC insulin in the diabetes group), altered gastrointestinal anatomy, hepatic or renal dysfunction, claustrophobia, MRI contraindications, or any condition likely to impair adherence with study procedures or data integrity.

### Pre-visit standardisation

**Fig. 1:**
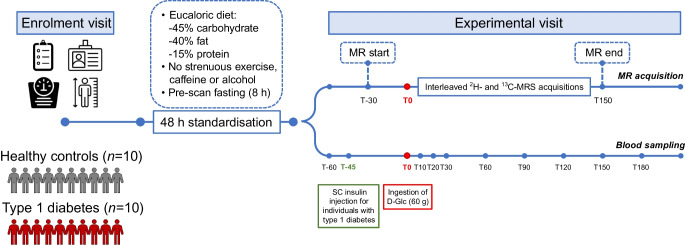
Study procedures. Prior to the experimental visit, participants underwent a 48 h standardisation protocol (refraining from strenuous exercise, caffeine and alcohol, while adhering to a eucaloric diet with defined macronutrient contributions). Metabolic imaging was performed at baseline under fasting conditions and for 150 min following ingestion of 60 g D-Glc. Blood sampling was conducted at multiple timepoints before, during and after MR acquisition (T−60, T0, T10, T20, T30, T60, T90, T120, T150, T180). After D-Glc intake, DMI and ^13^C-MRS were interleaved to acquire D-Glc and glycogen signals until T150. Participants with type 1 diabetes were administered SC insulin approximately 45 min before D-Glc intake (T−45). Icons are from https://thenounproject.com/

Prior to the experimental visit, participants followed a 48 h standardisation protocol [21] (Fig. 1) comprising adherence to a eucaloric diet and abstention from strenuous exercise, alcohol and caffeine. Energy requirements were estimated using the Harris–Benedict equation [23], with a physical activity factor of 1.3 (reflecting sedentary status), and macronutrient distribution according to Swiss dietary habits (45% carbohydrates, 15% protein, 40% fat) [24]. To ensure metabolic stability, participants with type 1 diabetes were instructed to report any clinically significant hypoglycaemia (capillary blood glucose <3.0 mmol/l) during the 48 h period, which would result in visit postponement by at least 48 h.

### Experimental visit

Following an 8 h overnight fast, participants attended the experimental visit, and underwent intravenous cannulation for blood sampling. The study protocol comprised postprandial assessment periods of 150 min for MR and 180 min for the blood sampling protocol. Participants with type 1 diabetes administered their calculated insulin dose to cover the ingested 60 g glucose using their individual carbohydrate-to-insulin ratio, with additional correction insulin based on current glucose levels, approximately 45 min before MRI (T−45). This constraint was due to the ultra-high magnetic field strength environment, requiring the removal of all metallic and electronic devices for insulin injection. The time lag of approximately 45 min was due to the need to acquire a baseline sample and leave enough time for the MR calibration, without repositioning the coil. Pump users had their pump removed and additionally received SC insulin detemir (Levemir, Novo Nordisk) to replace their basal insulin delivery rate over the subsequent 180 min. Participants ingested 60 g [6,6′-^2^H₂]-glucose (D-Glc, 99% purity; EURISOTOP, the European subsidiary of Cambridge Isotope Laboratories) dissolved in 200 ml water (time point T0). Venous blood samples were collected at T−60, T0, T10, T20, T30, T60, T90, T120, T150 and T180 min for analysis of plasma glucose concentration, glucose isotopic enrichment and insulin levels. Glucagon concentrations were assessed at selected time points (T0, T10, T30, T60, T120 and T180).

### Metabolic imaging protocol and data processing

Metabolic imaging was conducted on a 7 T Terra MR scanner (Siemens Healthineers). Participants were positioned supine, with a triple-tuned (^1^H, ^2^H, ^13^C) surface radiofrequency coil (Rapid Biomedical, Rimpar, Germany) placed over the right upper abdomen. An interleaved approach was employed to assess hepatic glucose metabolism: ^1^H-MRS was used for anatomical imaging; ^2^H-MRS (DMI) was performed to quantify the tissue content of D-Glc; and ^13^C-MRS was conducted to measure glycogen tissue content at ^13^C natural abundance [13].

For DMI, hepatic D-Glc concentrations were quantified by averaging signals from six central voxels selected manually (13.7 × 13.7 × 18.3 mm^3^), ensuring sufficient distance from larger vessels. The initial signal of natural-abundance mono-^2^H-labelled water served as the internal reference for glucose quantification, assuming a hepatic mono-^2^H-labelled water concentration of 8.94 mmol/l [11]. Thus, this measure represents the glucose concentration in a region of the liver consisting exclusively of hepatocytes and small vessels (sinusoids), and the concentration refers to mmol D-Glc per selected volume.

For ^13^C-MRS, liver glycogen content was quantified using phantom-derived reference data with additional corrections applied for coil-to-liver distance, coil loading effects, T₁ relaxation and nuclear Overhauser enhancement [21]. Technical details are provided elsewhere [13].

### Bioanalytical assays

Total plasma glucose concentration was determined using duplicate measurements taken using an Accu-Chek Inform II meter (Roche Diagnostics), and final values were calculated as the mean of the duplicates. To distinguish between different types of insulin (human insulin and various insulin analogues), insulin concentrations were quantified using LC-MS [25]. Glucagon was measured using a sequential sandwich immunoassay (no. 10-1271-01; Mercodia), with an additional washing step to reduce non-specific binding. Plasma D-Glc isotopic enrichment was measured using GC-MS in positive chemical ionisation mode (GC-CI/MS) using a 6890 gas chromatograph and a 5973 mass spectrometer (both Agilent).

### Differentiation of glucose sources

To differentiate between exogenous (orally ingested D-Glc) and endogenous glucose sources, molar ratios of D-Glc and protonated plasma glucose were corrected for tracer purity (99%) and natural abundance [22].

### Mathematical modelling of glucose–insulin interactions

The single tracer oral minimal model [26] was used to estimate the rate of appearance of D-Glc from the meal (*R*_aMeal_), endogenous glucose production (EGP) and the rate of glucose disposal (*R*_d_). *R*_aMeal_ was described using a model of gastrointestinal glucose absorption [27], enabling estimation of gastric retention (GR). The model provides estimates of glucose effectiveness for both glucose production and disposal (GE^Production^ and GE^Disposal^), as well as production and disposal insulin sensitivity indices (SI^Production^ and SI^Disposal^). Additionally, the following metabolic outcomes were computed: incremental AUC (iAUC) over 60 and 180 min (iAUC_0–60min_ and iAUC_0–180min_) for *R*_aMeal_ normalised to the ingested glucose dose (iAUC[*R*_aMeal_]/dose); iAUC of *R*_d_ normalised to its basal value (iAUC[*R*_d_/*R*_dBasal_]); iAUC of EGP normalised to its basal value (iAUC[EGP/EGP_Basal_], i.e. EGP suppression); and the time to 50% gastric emptying (T_50_GR). Model parameters were estimated by minimising the residual sum of squares between model-predicted D-Glc and endogenous glucose concentration profiles and the respective experimental data, using plasma insulin concentration as a known input, and assuming disposal glucose effectiveness at zero insulin (GEZI^Disposal^), fixed to a population value due to parameter identifiability issues [26]. Additional methodological details for the modelling approach are provided elsewhere [14].

For the type 1 diabetes group, some adjustments were needed to properly describe glucose–insulin interactions. First, to accommodate non-steady-state conditions at T0, measured fast-acting insulin (aspart or lispro) concentrations were described using a SC insulin absorption model [28], incorporating known bolus timing and dosing information to estimate individual insulin pharmacokinetic parameters. To account for additional long-acting insulin exposure, the active fraction of the long-acting insulin was superimposed, generating a composite total active insulin profile for subsequent glucose modelling, by applying established free-active fractions from the literature: 100% for insulin glargine [29, 30], 25% for insulin detemir [31] and 2.85% for insulin degludec [30]. In addition, to account for altered glucose effectiveness in individuals with diabetes, GEZI^Disposal^ was proportionally adjusted using literature information [26, 32, 33]. Finally, in contrast to the healthy population, GE^Production^ was too small to be reliably quantified using the present dataset in all individuals with type 1 diabetes, and was therefore fixed to zero. This parameter condenses the combined effect of glucose and portal insulin (direct effect) on glucose production, as extensively detailed in the original work in which this model was presented [34].

### Statistical analysis

The primary aim of this analysis was to characterise postprandial trajectories of hepatic D-Glc and glycogen signals, with a focus on identifying both between-group differences and within-group heterogeneity in type 1 diabetes. Given the absence of prior data in this specific experimental context, a target sample size of ten participants per group was established. Secondary analyses included characterisation of postprandial glucose–insulin interaction dynamics, accounting for potential differences in insulin and glucagon exposure profiles. Group differences were assessed using independent two-sample *t* tests, with results presented as means ± SD with 95% CI. Where indicated, SEM is reported. Statistical significance was defined as a two-sided *p* value <0.05. All statistical analyses were performed using MATLAB [35].

## Results

### Study participants

Participant characteristics are summarised in Table 1. Age, gender and BMI were similar between groups. The type 1 diabetes group exhibited long-standing type 1 diabetes with good glycaemic control, with mean HbA_1c_ of 50.1±8.9 mmol/l (6.7±0.8%) and a mean insulin requirement of 0.46±0.17 U kg^–1^ 24 h^–1^. Four participants used insulin aspart (Novorapid, Novo Nordisk), five used faster-acting insulin aspart (Fiasp, Novo Nordisk) and one used insulin lispro (Humalog, Eli Lilly); individuals on multiple daily injections (MDI) received either insulin glargine (Lantus, Sanofi) or degludec (Tresiba, Novo Nordisk) as their basal insulin. Participants with diabetes were administered 7.8±2.3 IU fast-acting insulin 46.2±6.3 min before D-Glc ingestion. The study sample was broadly representative of the young Swiss population in terms of sociodemographic characteristics. However, the included individuals with type 1 diabetes had comparatively favourable metabolic profiles (e.g. lower HbA_1c_) and the study was not designed for multi-ethnic recruitment.

**Table 1:** Baseline characteristics

		Individuals with type 1 diabetes				
Characteristic	Healthy control participants	All	Subgroup 1	Subgroup 2	*p* value^a^	*p* value^b^
Demographic variables						
Age (years)	36.4±10.0	38.0±9.8	38.7±8.1	37.0±13.4	0.72	0.83
Gender (female/male)	5/5	5/5	4/2	1/3		
Body weight (kg)	81.8±12.5	81.5±12.9	77.5±15.9	87.5±0.8	0.96	0.19
Height (m)	1.76±0.09	1.72±0.07	1.70±0.07	1.76±0.07	0.29	0.23
BMI (kg/m^2^)	26.3±3.0	27.4±3.4	26.7±4.0	28.4±2.1	0.44	0.42
Diabetes variables						
HbA_1c_ (mmol/mol)		50.1±8.9	48.5±9.2	52.5±9.3		0.52
HbA_1c_ (%)		6.7±0.8	6.6±0.8	6.9±0.9		0.52
Diabetes duration (years)		15.8±10.8	18.7±9.7	11.5±12.4		0.37
Total daily insulin dose (U/24 h)		37.3±15.7	32.4±15.8	44.6±14.2		0.24
Total daily insulin dose (U kg^–1^ 24 h^–1^)		0.46±0.17	0.42±0.18	0.51±0.16		0.46
Insulin treatment modality^c^		5/3/2	4/1/1	1/2/1		
Experimental details						
D-Glc ingestion period (min)	9.0±3.4	10.4±4.8	9.3±4.8	12.0±5.2	0.46	0.44
Insulin bolus dose for the D-Glc load (U)		7.8±2.3	8.2±2.1	7.4±2.9		0.65
Timing of the insulin bolus (min)		−46.2±6.3	−45.7±5.9	−48.0±9.9		0.80

Data for continuous variables are means ± SD; data for categorical variables are *n*

^a^*p* value for comparison of healthy control participants (*n*=10) vs those with type 1 diabetes (*n*=10), calculated using two-sample *t* tests

^b^*p* value for comparison of subgroup 1 (*n*=6) vs subgroup 2 (*n*=4), calculated using two-sample *t* tests

^c^Insulin treatments are: pump/MDI-degludec/MDI-glargine

While the metabolic trajectories were uniform in healthy control participants, distinct trajectories were observed for individuals of the type 1 group for several variables. Hierarchical clustering based on iAUCs of plasma and hepatic D-Glc revealed two distinct type 1 diabetes subgroups (see electronic supplementary material [ESM] Methods and ESM Fig. 1). Subgroup 1 comprised six participants; subgroup 2 included four. The clinical characteristics of the two subgroups are reported in Table 1.

**Fig. 2:**
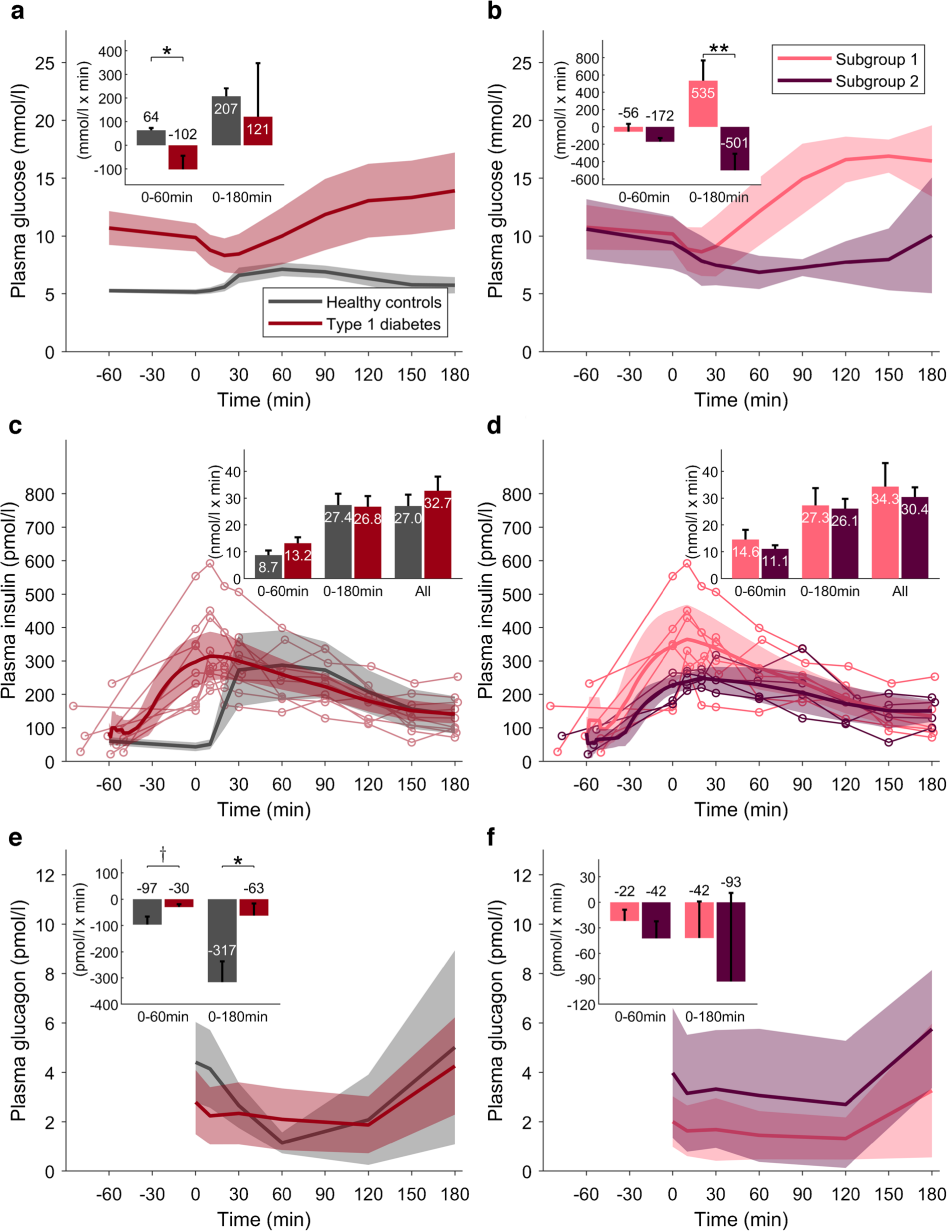
Measured plasma glucose and gluco-regulatory hormone profiles. Time courses are reported as the population average (continuous lines) with 95% CI (shaded areas) (*n*=10 for both groups). Insets: iAUC reported as mean and SEM bars: ^†^*p*<0.1; *p<0.05; **p<0.01; (**a**, **b**) Total plasma glucose concentrations. (**c**, **d**) Plasma insulin concentrations. (**e**, **f**) Plasma glucagon concentration. (**a**, **c**, **e**) Comparison of healthy control participants vs individuals with type 1 diabetes. (**b**, **d**, **f**) Subgroup analysis for subgroups 1 and 2. In (**c**) and (**d**), the individual data for those with type 1 diabetes are shown, and the population curve refers to the model-derived continuous profiles (see main text for details). The iAUC is also reported for the entire duration of the experiment (labelled ‘All’), referring to the interval from T−60 to T180

All participants completed the study, but one healthy participant was excluded due to issues with model prediction and parameter estimation. In the type 1 diabetes group, the model of SC fast-acting insulin kinetics accurately captured the observed insulin exposure (Fig. 2c, d) and parameters of glucose–insulin interactions were estimated with adequate precision.

### Plasma glucose and hormonal responses

Fasting plasma glucose was higher in individuals with type 1 diabetes compared with healthy control participants (10.7 ± 2.3 vs 5.2 ± 0.4 mmol/l; *p*<0.001) (Table 2). The maximum postprandial glucose concentration was also higher in the type 1 diabetes group (15.7 ± 4.2 vs 7.5 ± 0.8 mmol/l, *p*<0.001). Within the type 1 diabetes cohort, subgroup 1 exhibited a higher peak glucose concentration than subgroup 2 (*p*=0.004). Following administration of the SC insulin bolus, all type 1 diabetes participants showed an initial decline in glucose levels (iAUC_0–60_ [mean ± SD]=−102 ± 182 mmol/l × min, Fig. 2a); this was slightly more pronounced in subgroup 2 (Fig. 2b). Although initial insulin exposure was higher in the type 1 diabetes group due to early SC insulin administration, total insulin exposure over the experimental period was not significantly different between groups (32.7 ± 16.7 vs 27.0 ± 13.4 nmol/l × min; *p*=0.41) (Table 2). Insulin level trajectories are illustrated in Fig. 2c, d.

**Table 2:** Basal concentrations, peak or nadir concentrations and time of measured metabolic and endocrine signals

			Individuals with type 1 diabetes				
Variable		Healthy control participants	All	Subgroup 1	Subgroup 2	*p* value^a^	*p* value^b^
Plasma							
Total glucose							
Basal (mmol/l)		5.2±0.4	10.7±2.3	10.8±2.4	10.6±2.6	<0.001	0.92
Peak (mmol/l)		7.5±0.8	15.7±4.2	18.4±2.2	11.5±2.5	<0.001	0.004
Peak time (min)		84.1±54.4	135.4±60.4	135.3±40.9	135.5±90.3	0.06	1
Exogenous glucose (D-Glc)							
Peak (mmol/l)		5.2±1.2	10.3±3.4	12.6±1.7	6.7±1.6	<0.001	0.001
Peak time (min)		126.1±48.4	150.3±37.3	145.2±34.4	158.0±45.3	0.23	0.65
Endogenous glucose							
Nadir (mmol/l)		1.2±0.6	4.5±2.1	5.0±2.2	3.9±2.0	<0.001	0.46
Nadir time (min)		171.2±14.4	162.5±21.2	×160.5±24.5	165.5±17.9	0.30	0.72
Insulin							
Basal (pmol/l)		43.4±20.5	74.9±61.0	88.5±75.9	54.3±24.8	0.15	0.34
Peak (pmol/l)		366±192	324±112	370±125	255±35	0.56	0.08
Peak time (min)		81.2±47.1	18.5±18.9	13.2±11.4	26.5±26.8	0.002	0.40
AUC_0–60_ (nmol/l × min)		8.7±5.5	13.2±7.0	14.6±8.8	11.1±2.7	0.13	0.40
AUC_0–180_ (nmol/l × min)		27.4±13.4	26.8±12.6	27.3±15.8	26.1±7.3	0.92	0.88
AUC_T−60–180_ (nmol/l × min)		27.0±13.4	32.7±16.7	34.3±21.5	30.4±7.2	0.41	0.70
Mean (pmol/l)		172.0±77.6	210.3±57.5	230.4±64.2	180.3±32.4	0.23	0.14
Glucagon							
T0 (pmol/l)		4.41±2.57	2.80±2.05	2.01±1.26	3.98±2.63	0.14	0.24
Nadir (pmol/l)		1.05±0.66	1.69±1.67	1.15±1.05	2.51±2.24	0.28	0.32
Nadir time (min)		63.0±22.1	55.0±51.5	51.7±57.4	60.0±49.0	0.66	0.81
AUC_0–60_ (pmol/l × min)		−96.9±97.2	−30.2±35.1	−21.9±32.3	−42.5±40.3	0.07	0.43
AUC_0–180_ (pmol/l × min)		−316.6±253.6	−62.6±146.4	−42.1±105.4	−93.5±209.0	0.02	0.67
Mean (pmol/l)		2.65±2.03	2.45±1.98	1.77±1.49	3.46±2.40	0.82	0.27
Imaging (DMI and ^13^C-MRS)							
Hepatic exogenous glucose (D-Glc)	Peak (mmol/l)	3.0±0.8	4.7±2.0	6.1±0.8	2.7±0.9	0.02	<0.001
	Peak time (min)	91.7±32.2	131.3±30.2	131.4±21.8	131.0±44.1	0.01	0.99
Hepatic glycogen	Basal (mmol/l)	230±46	289±87	274±86	312±96	0.08	0.54

Data are means ± SD

Peaks and nadirs are defined as the maximum and minimum values, respectively, attained during the experimental period, excluding the T−60 sample

^a^*p* value for comparison of healthy control participants (*n*=10) vs those with type 1 diabetes (*n*=10), calculated using two-sample *t* tests

^b^*p* value for comparison of subgroup 1 (*n*=6) vs subgroup 2 (*n*=4), calculated using two-sample *t* tests

In both groups, glucagon levels declined following D-Glc ingestion and subsequently rose near the end of the observation period (Fig. 2e, f). Net suppression of glucagon after T0 was less pronounced in individuals with diabetes (iAUC_0–180_=−63 ± 146 vs −317 ± 254 pmol/l × min; *p*=0.016). Plasma glucagon concentrations before administration of D-Glc were not significantly different between individuals with type 1 diabetes and healthy control participants (2.80 ± 2.05 vs 4.41 ± 2.57 pmol/l; *p*=0.14); however, these values do not represent true basal levels in the diabetes group as sampling was performed after the insulin bolus.

### Plasma D-Glc and endogenous glucose profiles

**Fig. 3:**
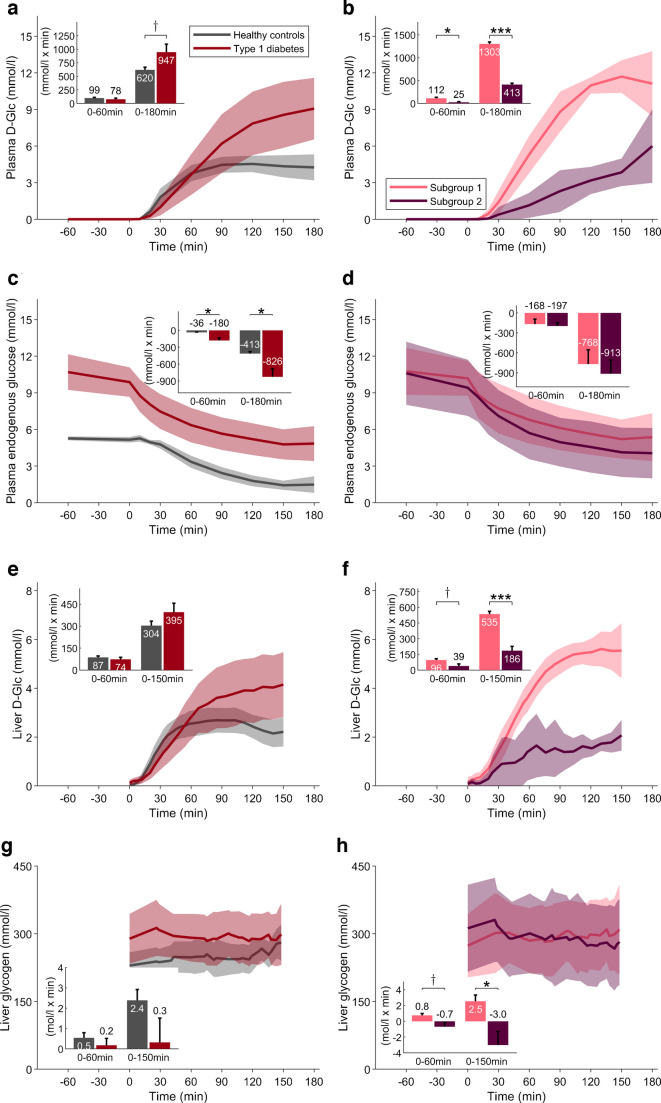
Measured glucose and glycogen profiles. Time courses are reported as the population average (continuous lines) with 95% CI (shaded areas) (*n*=10 for both groups). Insets: iAUC reported as mean and SEM bars: ^†^*p*<0.1; *p<0.05; ***p<0.001. (**a**, **b**) Plasma D-Glc concentration. (**c**, **d**) Plasma endogenous glucose concentration. (**e**, **f**) Liver D-Glc concentration. (**g**, **h**) Liver glycogen concentration. (**a**, **c**, **e**,** g**) Comparison of healthy control participants vs individuals with type 1 diabetes. (**b**, **d**, **f**,** h**) Subgroup analysis for subgroup 1 and 2

Plasma D-Glc reached a higher peak concentration in individuals with type 1 diabetes compared with healthy control participants (10.3 ± 3.4 vs 5.2 ± 1.2 mmol/l, *p*<0.001) (Table 2 and Fig. 3a), and an almost twofold difference was observed in subgroup 1 vs subgroup 2 (*p*=0.001). The profiles in the two subgroups were markedly different, with a reduced appearance of D-Glc in subgroup 2 (*p*=0.012 and *p*<0.001, for iAUC after 60 and 180 min, respectively) (Fig. 3b).

For plasma endogenous glucose, the time to reach nadir values was similar between groups (162.5 ± 21.2 vs 171.2 ± 14.4 min; *p*=0.30). Minimum endogenous glucose levels (nadir) remained higher in individuals with type 1 diabetes than in healthy control participants (4.5 ± 2.1 vs 1.2±0.6 mmol/l; *p*<0.001) (Table 2), although the absolute decrease from baseline was greater in the type 1 diabetes group (Fig. 3c). Both type 1 diabetes subgroups exhibited comparable initial levels and time course trajectories for plasma endogenous glucose (Fig. 3d).

### Liver imaging for glucose and glycogen signals

Hepatic D-Glc profiles exhibited similar time course patterns to those for plasma D-Glc (Fig. 3e, f). Peak hepatic D-Glc concentrations were greater in individuals with type 1 diabetes than healthy control participants (4.7±2.0 vs 3.0±0.8 mmol/l; *p*=0.02) (Table 2), and this effect was only driven by subgroup 1. Subgroup analysis within the type 1 diabetes cohort revealed a more than twofold difference in the maximum value for hepatic D-Glc concentration (*p*<0.001). In healthy control participants, hepatic D-Glc levels declined after 90 min, coinciding with an increase in hepatic glycogen (iAUC_0–180_=2.4 mol/l × min), but this pattern was absent in participants with type 1 diabetes (Fig. 3g). However, subgroup analysis within the type 1 diabetes cohort revealed distinct postprandial glycogen trajectories, with net accumulation in subgroup 1 and net depletion in subgroup 2 (iAUC_0–180_=2.5 vs –3.0 mol/l × min; *p*=0.04) (Fig. 3h). Baseline hepatic glycogen levels were slightly higher in individuals with diabetes compared with healthy control participants, although not significantly (289±87 vs 230±46 mmol/l, *p*=0.08) (Table 2 and Fig. 3g).

### Parameters of glucose–insulin interaction

**Fig. 4:**
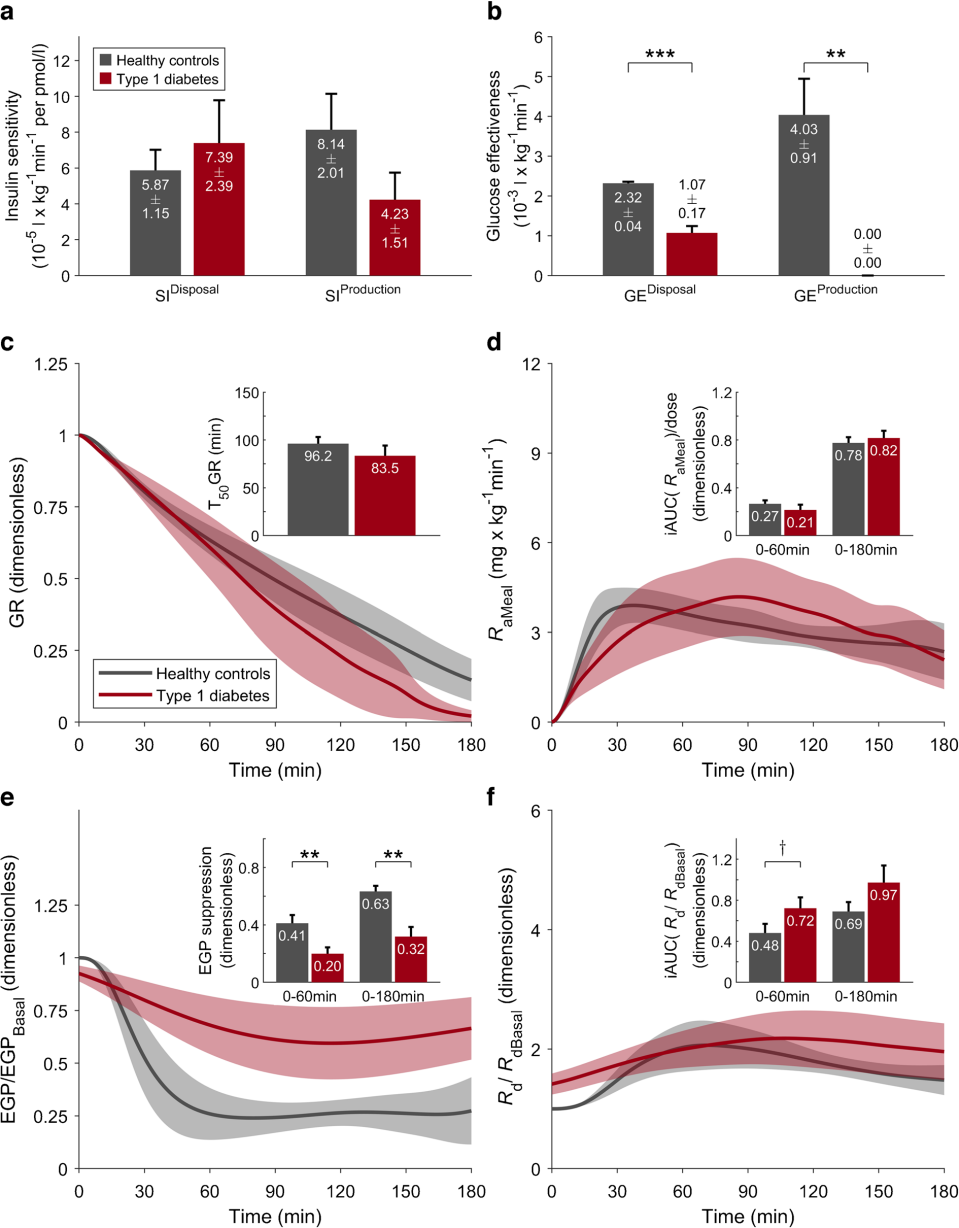
Estimated model parameters and model-derived fluxes. Comparisons between healthy control participants (*n*=9, one participant was excluded from the modelling analysis) and individuals with type 1 diabetes (*n*=10): ^†^*p*<0.1; **p<0.01; ***p<0.001. (**a**, **b**) Values are means ± SEM. (**c**–**f**) Time courses are shown as population average (continuous lines) with 95% CI (shaded areas). (**a**) Disposal and production insulin sensitivity. (**b**) Disposal and production glucose effectiveness. (**c**) Postprandial time course of GR; inset: T_50_GR, time to half stomach emptying. (**d**) *R*_aMeal_; inset: fraction of the ingested D-Glc that has appeared in plasma after 60 and 180 min. (**e**) EGP, normalised to basal; inset: fractional EGP suppression. (**f**) *R*_d_/*R*_dBasal_; inset: fractional increase of rate of disposal. All insets show mean and SEM

SI^Disposal^ was comparable between the type 1 diabetes and healthy control groups, whereas SI^Production^, which mainly reflects hepatic insulin sensitivity, was nearly halved in individuals with type 1 diabetes, but this difference was not statistically significant (*p*=0.14, Fig. 4a). Within the type 1 diabetes group, SI^Disposal^ was more than threefold greater in subgroup 2, but this difference did not reach statistical significance (*p*=0.20). GE^Disposal^ was significantly reduced in type 1 diabetes, due to the lower population value for GEZI^Disposal^ [26] (Fig. 4b).

**Fig. 5:**
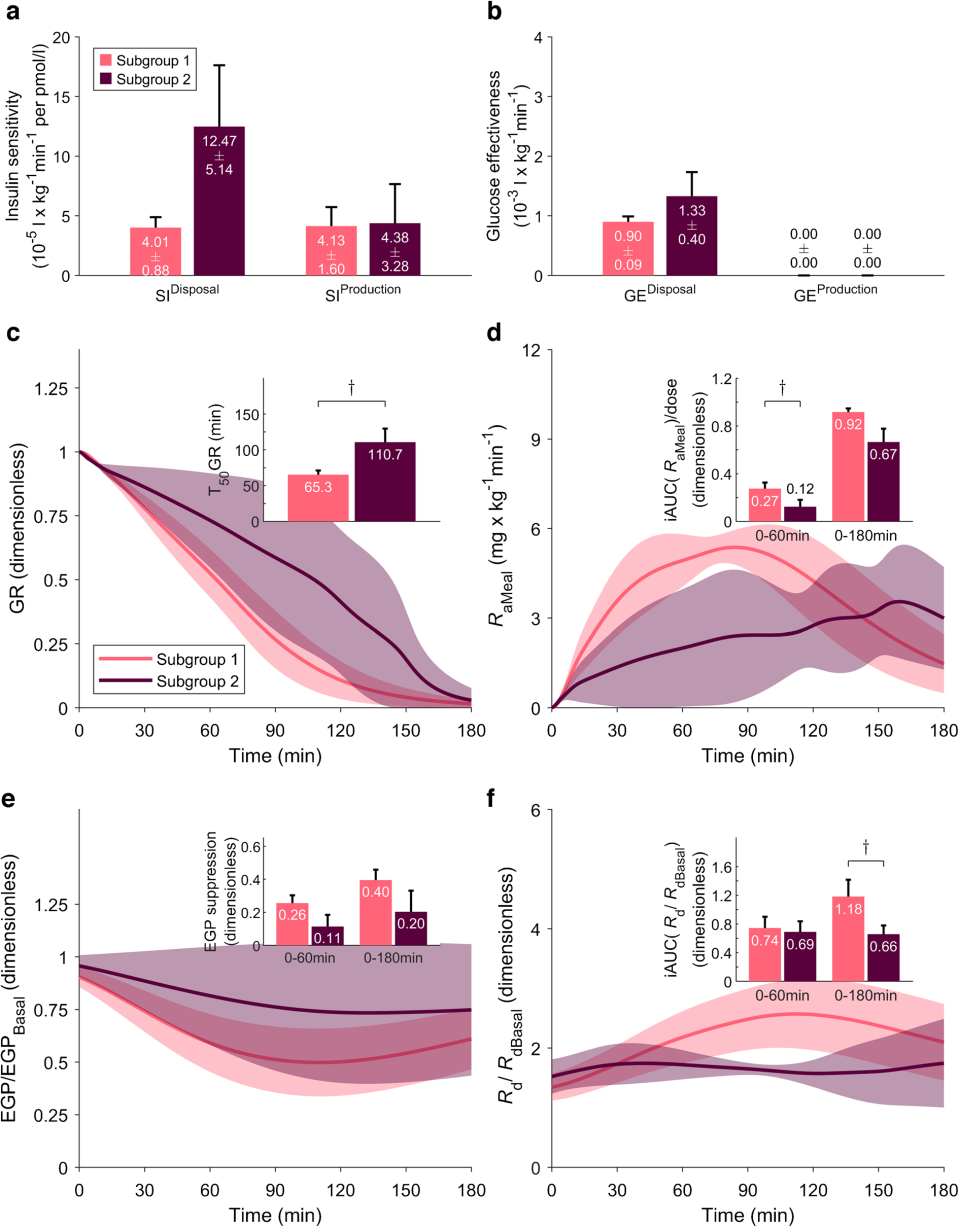
Estimated model parameters and model-derived fluxes. Comparisons between subgroup 1 (*n*=6) and subgroup 2 (*n*=4): ^†^*p*<0.1. (**a**, **b**) Values are means ± SEM. (**c**–**f**) Time courses are shown as population average (continuous lines) with 95% CI (shaded areas). (**a**) Disposal and production insulin sensitivity. (**b**) Disposal and production glucose effectiveness. (**c**) Postprandial time course of GR; inset: T_50_GR, time to half stomach emptying. (**d**) *R*_aMeal_; inset: fraction of the ingested D-Glc that has appeared in plasma after 60 and 180 min. (**e**) EGP, normalised to basal; inset: fractional EGP suppression. (**f**) *R*_d_/*R*_dBasal_; inset: fractional increase of rate of disposal. All insets show mean and SEM

Model-predicted GR and *R*_aMeal_ were similar between populations (Fig. 4c, d), indicating no substantial differences in glucose absorption kinetics between the groups. However, considerable heterogeneity was observed within the type 1 diabetes cohort: Subgroup 1 exhibited almost twofold faster gastric emptying compared with subgroup 2 (T_50_GR=65.3 vs 110.7 min; *p*=0.09) (Fig. 5c), paralleled by a higher iAUC_0–60_(*R*_aMeal_)/dose (*p*=0.09, Fig. 5d), suggesting a tendency towards more rapid glucose absorption, although neither difference reached statistical significance.

We observed a marked difference in EGP suppression between individuals with type 1 diabetes and healthy control participants (Fig. 4e), with suppression in the type 1 diabetes group reduced by approximately half at both 60 and 180 min compared with healthy control participants (*p*=0.01 and *p*=0.001, respectively). This finding is consistent with the numerically lower SI^Production^ in the type 1 diabetes cohort. Subgroup analysis revealed a distinct pattern: compared with subgroup 1, EGP suppression in the first hour was more than halved in subgroup 2, but not significantly (0.11 vs 0.26: *p*=0.15) (Fig. 5e).

The postprandial increase in glucose disposal (iAUC[*R*_d_/*R*_dBasal_]) (Fig. 4f) showed slightly different patterns in individuals with type 1 diabetes compared with healthy control participants, probably reflecting differences in the temporal profile of insulin exposure. Subgroup analysis within the type 1 diabetes cohort indicated a lower postprandial glucose disposal capacity in subgroup 2, but this difference was not statistically significant (0.66 vs 1.18; *p*=0.087) (Fig. 5f), which may correspond to the lower *R*_aMeal_ observed in this subgroup.

## Discussion

In this study, we integrated a novel hepatic metabolic imaging protocol with whole-body deuterium glucose dilution to characterise postprandial glucose metabolism in individuals with type 1 diabetes and healthy control individuals. This integrated approach enabled simultaneous assessment of liver-specific glucose fluxes and systemic glucose–insulin homeostasis, allowing us to elucidate the differences in postprandial glucose handling between normal physiology and adults with well-managed type 1 diabetes receiving SC insulin therapy.

The key findings of this study were threefold. First, despite similar systemic insulin exposure, EGP was less effectively suppressed following a glucose load in individuals with type 1 diabetes compared with healthy control participants similar in age, BMI and gender distribution, confirming altered hepatic glucose regulation in this population, even with optimal SC insulin therapy. Second, although the postprandial increase in hepatic glycogen was diminished in type 1 diabetes, overall glycogen storage capacity after dietary standardisation was preserved. Third, despite comparable clinical characteristics among individuals with type 1 diabetes, we identified distinct patterns of postprandial glucose regulation, probably reflecting heterogeneity in gastric emptying or other splanchnic glucose regulatory mechanisms.

Modelling analysis in healthy control participants revealed a suppression of EGP of 63% over 3 h, in line with double tracer-derived literature results [36]. In this study, we interpret the diminished suppression of EGP in individuals with type 1 diabetes primarily in the context of non-physiological SC insulin administration. It is well established that hepatic glucose suppression is regulated chiefly by the direct action of insulin on the liver, and that increasing systemic insulin levels cannot compensate for reduced hepatic insulin exposure [4]. In other words, the lack of porto-systemic insulin gradient is reflected in a reduction of insulin’s efficacy in suppressing EGP, consistent with the finding that SI^Production^ is halved in individuals with type 1 diabetes compared with control participants.

The absorption of carbohydrates results in increases in plasma glucose concentrations, which usually starts about 10–20 min after the start of a meal, but this effect can vary based on a number of factors (e.g. meal composition, duration of diabetes, autonomic dysfunction and alterations in the microbiota of the upper gastrointestinal tract) [37]. Although impaired postprandial glucagon suppression, and even paradoxical increases, have been reported in type 1 diabetes [38, 39], our observations do not support glucagon abnormalities as a key driver of insufficient EGP suppression. While prandial insulin was administered exceptionally early (mean 46.2 min earlier) in this study to meet safety requirements in the MR environment, our findings demonstrate that early SC insulin delivery – even with faster insulin absorption profiles – is unlikely to fully normalise EGP suppression. In contrast, approaches that preferentially increase hepatic insulin exposure, such as hepatoselective insulin analogues, insulin delivery routes that increase hepatic insulin exposure (e.g. intraperitoneal infusion) or intraportal islet transplantation, may allow more effective normalisation of hepatic glucose regulation. However, their clinical use remains limited [4, 6, 40, 41].

The glycogen levels observed prior to D-Glc intake, i.e. after 48 h of standardised diet, activity and absence of clinically relevant hypoglycaemia, indicate that individuals with type 1 diabetes are not generally impaired in their overall capacity to store glycogen, provided glycaemic control is adequate. This finding is consistent with previous research on glycogen storage in type 1 diabetes [42], but contrasts with the observed postprandial glycogen accumulation patterns in our study, which were evident in healthy control participants but absent in type 1 diabetes. Although this finding was primarily driven by subgroup 2, the overall observation of modestly elevated baseline glycogen supports a greater reliance on the indirect pathway for hepatic glycogen synthesis in individuals with type 1 diabetes, as previously demonstrated [43]. This pathway is distinct from the direct route, in which glucose taken up by the liver is directly incorporated into glycogen.

Unexpectedly, we identified two distinct subgroups of type 1 diabetes based on postprandial glucose trajectories, despite no overt clinical differences. While we do not consider this as evidence for distinct pathophysiological subtypes in type 1 diabetes, and it is possibly even a product of random chance, these subgroups possibly represent the natural heterogeneity that is inherent within a broader phenotypic spectrum, providing a valuable framework for exploring the mechanisms that drive inter-individual differences in postprandial glucose metabolism. Subgroup 1 exhibited elevated plasma and hepatic D-Glc levels compared with subgroup 2, which resulted in lower SI^Disposal^. Furthermore, glycogen levels decreased in subgroup 2 but increased in subgroup 1. We interpret these differential responses as variations in exogenous glucose appearance kinetics, probably attributable to differences in gastric emptying kinetics (specifically, a faster emptying rate in subgroup 1 than subgroup 2) and, consequently, differences in glucose absorption. Slowed gastric emptying delays glucose appearance in the portal circulation, thereby limiting glucose availability for postprandial glycogen synthesis. Evidence shows that gastric emptying kinetics, measured using gold-standard techniques (scintigraphy and ^13^C breath tests), are key to postprandial glucose levels in type 1 diabetes [44, 45]. Gastric emptying is highly variable among individuals with type 1 diabetes: both delayed and accelerated gastric emptying have been described [44–46]. Although no major subgroup differences in insulin and glucagon levels were evident, it is possible that additional, as yet unidentified neurohormonal factors [3, 47] released from the gut or within the portal circulation, in conjunction with differences in gastric emptying, contribute to the fine-tuned regulation of postprandial hepatic glucose metabolism. Furthermore, autonomic neuropathy (which was not quantified in this study) may be a source of alterations in gastric emptying kinetics and other aspects of postprandial metabolism. Together, these factors may help explain the distinct metabolic trajectories observed.

The strength of this study is the application of a novel hepatic imaging protocol with whole-body deuterium glucose dilution techniques to provide new quantitative insights into postprandial glucose metabolism in type 1 diabetes, including unexpected phenotypic variability. The type 1 diabetes and healthy control groups were similar in terms of age, gender and BMI, factors that are known to independently influence hepatic glucose regulation. Additionally, the pre-experimental standardisation period and the selection of participants with well-managed diabetes minimised confounding factors that could interfere with glucose regulation. Nonetheless, we acknowledge several limitations in this study. Imaging was conducted in a supine position, which is known to influence gastric emptying compared with the upright posture [48]. Additionally, the postprandial scanning period was limited to 150 min to minimise participant discomfort; however, this time frame may have been insufficient to fully capture the complete dynamics of postprandial metabolism following a 60 g oral glucose load [49]. One potential approach to address this limitation would be to reduce the D-Glc dose, which may enable observation of the full glucose trajectory within the scanning window. However, use of lower doses would compromise the detection of concomitant glucose-to-glycogen conversion using natural-abundance ^13^C-MRS.

The modest sample size, constrained by the resource-intensive nature of the experimental methodology, limited the statistical power, particularly for the subgroup analysis. Nevertheless, our findings provide clinically relevant insights into postprandial glucose metabolism in type 1 diabetes, establishing a foundation for future large-scale validation. Future studies should prioritise greater population diversity, incorporating individuals with suboptimal glucose management and evaluating sex-specific physiology, including attention to the type of contraceptive method and menstrual cycle phase at the time of metabolic assessments. Finally, there remains considerable scope for methodological refinement. While the interleaved DMI/^13^C-MRS approach used in this study provides novel, direct insights into postprandial hepatic glucose metabolism in vivo, interpreting the D-Glc signal from DMI is constrained by its composite nature, i.e. the vascular and hepatocellular signals are superimposed. In other words, DMI as a stand-alone method does not provide a direct measure of hepatocellular glucose uptake, unlike ^18^F-fluorodeoxyglucose positron emission tomography. To address this limitation, further methodological development is needed, including the refinement of kinetic models that estimate hepatic glucose disposal [50] and the implementation of advanced imaging acquisition schemes that are capable of distinguishing vascular from tissue signals. Furthermore, use of an extended DMI technique using a specialised deuterium body array coil [10] could track ^2^H-labelled glucose kinetics throughout the gastrointestinal tract, enabling direct quantification of gastric emptying.

In summary, this comparative study demonstrates that adults with well-managed type 1 diabetes exhibit distinct alterations in postprandial glucose metabolism compared with healthy control participants. Notably, we identified unexpected phenotypic variability within the type 1 diabetes cohort, probably reflecting differences in gastric emptying and/or glucose absorption kinetics. Although the interpretation and applicability of these results are limited by the sample size and the lack of gastric emptying assessment, our findings underscore the pressing need to elucidate the mechanisms governing postprandial glucose regulation at the splanchnic level in type 1 diabetes. The integration of advanced metabolic imaging with stable-isotope tracer modelling, as illustrated in this study, offers a promising framework for revealing the complex interplay among the gastrointestinal tract, portal circulation and hepatic signalling pathways. Deeper mechanistic insight into these regulatory processes may help explain individual variability in postprandial glucose management and support the development of personalised therapeutic strategies in type 1 diabetes, which is particularly relevant in the context of emerging incretin-based therapies and the evolution of fully automated closed-loop insulin delivery systems.

## Supplementary Information

Below is the link to the electronic supplementary material.

ESM1 (PDF 276 KB)

## Data Availability

The data that support the findings of this study are available from the corresponding author upon reasonable request.

## Abbreviations

D-Glc: [6,6′-^2^H_2_]-glucose
DMI: Deuterium metabolic imaging
EGP: Endogenous glucose production
EGP_basal_: Basal endogenous glucose production
FDG-PET: ^18^F-fluorodeoxyglucose-positron emission tomography
GE^Disposal^: Disposal glucose effectiveness
GE^Production^: Production glucose effectiveness
GEZI^Disposal^: Disposal glucose effectiveness at zero insulin
GR: Gastric retention
iAUC: Incremental AUC
iAUC_0–150_: Incremental AUC until *t*=150 min
iAUC_0–180_: Incremental AUC over the course of the experiment
iAUC_0–60_: Incremental AUC over the first hour
MDI: Multiple daily injections
MR: Magnetic resonance
MRS: Magnetic resonance spectroscopy
*R*_aMeal_: Rate of glucose appearance from the meal
*R*_d_: Rate of glucose disposal
*R*_dBasal_: Basal rate of glucose disposal
SC: Subcutaneous
SI^Disposal^: Disposal insulin sensitivity
SI^Production^: Production insulin sensitivity
T_50_GR: Time to 50% gastric retention
