# Supplementary material for: Novel deuterium metabolic imaging technique reveals distinct patterns of postprandial hepatic glucose homeostasis in individuals with type 1 diabetes and healthy control individuals: a case–control study
Source: Diabetologia. 2026 Feb 13;69(5):1301–16. doi: 10.1007/s00125-026-06677-7 (PMC13005865; doi:10.1007/s00125-026-06677-7)

ESM Methods: Hierarchical Clustering

An agglomerative hierarchical clustering approach was used to stratify the individuals with type 1 diabetes. The variable space was defined as the set of iAUCs in the intervals 0-60 min and 0-180 min, for plasma D-Glc and liver D-Glc. The selection of signals for clustering was performed by visual inspection of measured profiles. The Euclidean pairwise distance was computed between subjects based on these four values, the clusters were formed iteratively using the average linkage method. The optimal number of clusters was then selected via the silhouette score, allowing to account for both inter-cluster separation and intra-cluster cohesion.

ESM Figure 1. Representation of the hierarchical clustering procedure used to stratify individuals with type 1 diabetes into two subgroups. **(a)** Individual profiles of plasma exogenous glucose (D-Glc); **(b)** Individual profiles of liver exogenous glucose (D-Glc); **(c)** Dendrogram of the clustering tree; **(d)** Representation of the ten subjects (coloured by subgroup) in the space defined by $iAUC_{0-180}$(Plasma D-Glc) vs. $iAUC_{0-180}$(Liver D-Glc).

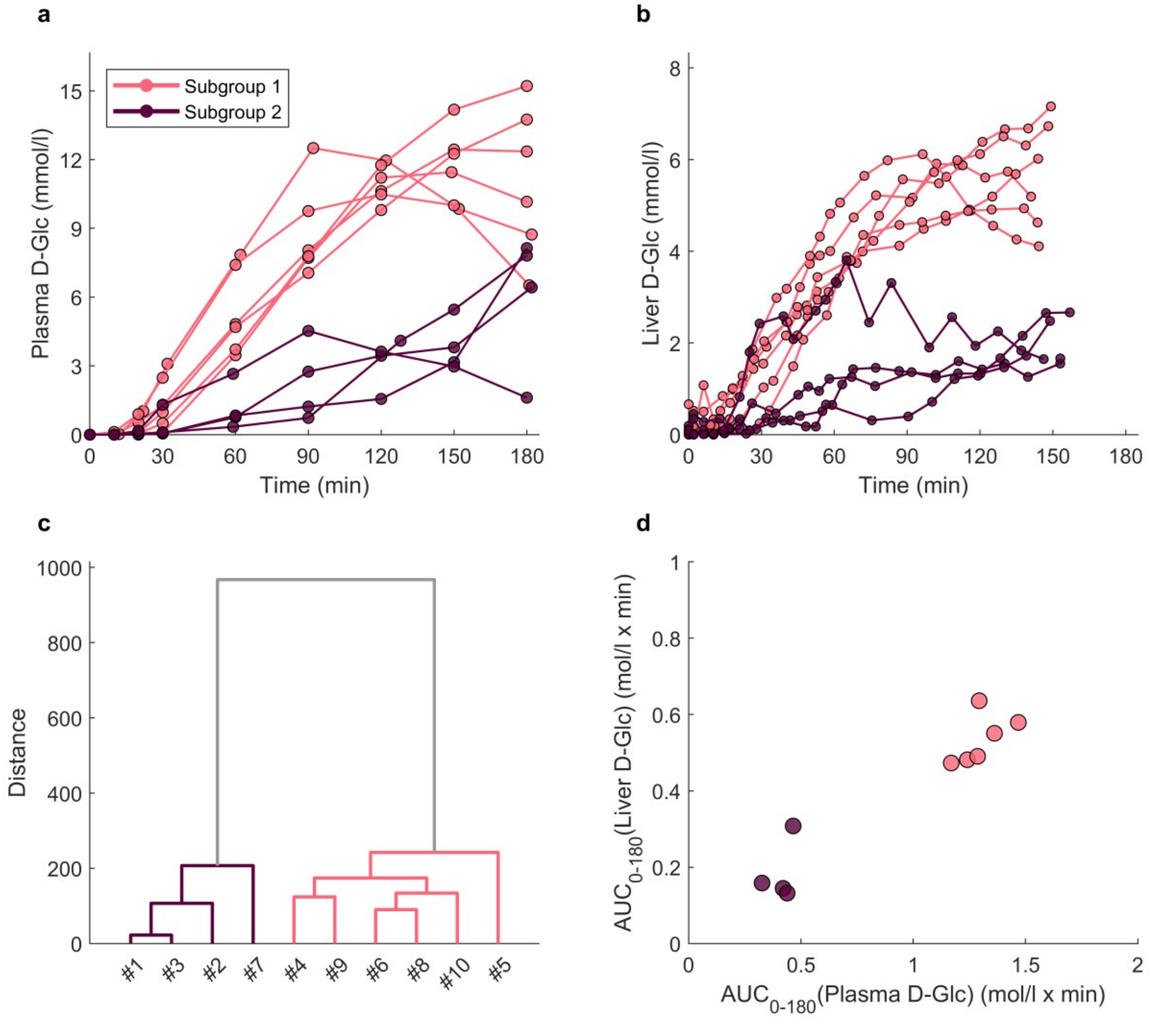

Supplement: Supplementary file 1 — ESM1 (PDF 276 KB) [file 125_2026_6677_MOESM1_ESM.pdf]
